# Cyclanilide Induces Lateral Bud Outgrowth by Modulating Cytokinin Biosynthesis and Signalling Pathways in Apple Identified via Transcriptome Analysis

**DOI:** 10.3390/ijms23020581

**Published:** 2022-01-06

**Authors:** Juanjuan Ma, Lingling Xie, Qian Zhao, Yiting Sun, Dong Zhang

**Affiliations:** College of Horticulture, Yangling Subsidiary Center Project of the National Apple Improvement Center, Northwest A&F University, Yangling, Xianyang 712100, China; mjj@nwafu.edu.cn (J.M.); 2019055163@nwafu.edu.cn (L.X.); zq1037356493@163.com (Q.Z.); 17835422544@163.com (Y.S.)

**Keywords:** cyclanilide, apple, branching, transcriptome, cytokinin

## Abstract

Cyclanilide (CYC), a plant growth regulator, is a potent shoot branching agent in apple. However, its mechanism remains unclear. The current study revealed that CYC treatment resulted in massive reprogramming of the axillary bud transcriptome, implicating several hormones in the response. We observed a marked increase (approximately 2-fold) in the level of zeatin riboside and a significant decrease (approximately 2-fold) in the level of abscisic acid (ABA). Zeatin metabolism gene cytokinin (CTK) oxidase 1 (*CKX 1*) was down-regulated at 168 h after CYC treatment compared with the control. Weighted gene co-expression network analysis of differentially expressed genes demonstrated the turquoise module clusters exhibited the highest positive correlation with zeatin riboside (r = 0.92) and the highest negative correlation with ABA (r = −0.8). A total of 37 genes were significantly enriched in the plant hormone signal transduction pathway in the turquoise module. Among them, the expressions of CTK receptor genes *WOODEN LEG* and the CTK type-A response regulators genes *ARR3* and *ARR9* were up-regulated. ABA signal response genes protein phosphatase 2C genes *ABI2* and *ABI5* were down-regulated in lateral buds after CYC treatment at 168 h. In addition, exogenous application of 6-benzylaminopurine (6-BA, a synthetic type of CTK) and CYC enhanced the inducing effect of CYC, whereas exogenous application of lovastatin (a synthetic type of inhibitor of CTK biosynthesis) or ABA and CYC weakened the promoting effect of CYC. These results collectively revealed that the stimulation of bud growth by CYC might involve CTK biosynthesis and signalling, including genes *CKX1* and *ARR3/9*, which provided a direction for further study of the branching promoting mechanism of CYC.

## 1. Introduction

Cyclanilide (CYC) is a plant growth regulator which is registered for use in cotton growth control at different development stages, but only in combination with other plant growth regulators, such as ethephon for defoliation [1]. CYC used alone can weaken apical dominance to enhance apple nursery stock lateral branching development [2], and the fast formation of lateral branches in apple is conducive to early and increased yields [2,3]. CYC is a potent branching agent in apple [4], but its mechanism remains unclear.

In plants, the inhibition of axillary bud outgrowth by the growing shoot apex is called apical dominance [5,6,7]. It has long been considered that auxin plays a central role in this phenotype. Since auxin does not enter axillary buds, it acts indirectly [8]. There is a lot of evidence supporting two non-mutually exclusive models for the indirect action of auxin on branching [9]. One is referred to as the “second messenger” model, where auxin in the main stem regulates the synthesis of cytokinins (CTKs) and strigolactones (SLs) that move up into the buds and control their activity. CTKs act as bud outgrowth inducers that antagonise auxin on branching [10,11]. SLs act as bud outgrowth repressors and enhance the inhibitory effect of auxin on branching [12]. The other is referred to as the “auxin canalization” model, where stem auxin influences the establishment of canalized auxin transport from the bud into the main stem [13].

Branching is a plastic trait which is influenced by hormones, developmental factors, nutrients and their complex interactions [5,6]. Among the hormones, indole-3-acetic acid (IAA), CTK and SL are not the only three hormones that have effects on plant branching. Gibberellin (GA), abscisic acid (ABA), jasmonic acid (JA), and brassinolide (BR) also play an important role in branching control in some plant species [14,15,16,17]. Sucrose is an early regulator of the key hormonal mechanisms controlling rose bud outgrowth [18] and sugar prevents auxin, and strigolactone pathways to promote bud outgrowth [19].

BRC1 (BRANCHED1) has been proposed to act as a central integrator of branching control belonging to the TB1/CYC/PCFs (TCP) family [20]. *BRC1* expression is related to bud inhibition in several species, and its transcript level can be up-regulated by SLs and down-regulated by CTKs [21]. In *Arabidopsis*, the BRANCHED1/HD-ZIP I cascade enhances ABA accumulation and triggers a hormone response, thus causing the suppression of branching [15].

The molecular mechanism of axillary bud growth regulation by CYC is still unclear in apple. In the current study, high-throughput RNA sequencing (RNA-seq) was used to identify the branching-related genes and probable pathways in apple after exogenous CYC treatment. To investigate the physiological basis of CYC on branching, hormone content and branching phenotype analysis were combined with the administration of various pharmacologic treatments. Overall, stimulation of bud growth by CYC may involve CTK biosynthesis and signalling, including genes *CKX1* and *ARR3/9*. The result provided a direction for further study of the branching promoting mechanism of CYC.

## 2. Results

### 2.1. Influence of CYC on Bud Outgrowth Patterns

**Figure 1:**
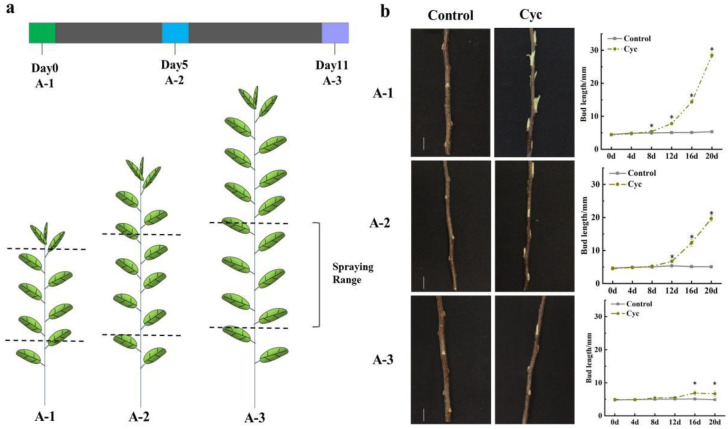
Apple bud phenotype and bud length of axillary buds subjected to cyclanilide application in 1-year apple seedlings. Scale bar = 2.0 cm. (**a**) treatment method; (**b**) bud phenotype and length. Cyc: cyclanilide. Data represent the mean ± SD (*n* = 15). Asterisk indicates significant differences based on the Tukey test (*p* < 0.05).

To evaluate the impact of CYC on axillary bud outgrowth in apple (*Malus domestica* Borkh), we treated the current-season’s ‘Fuji’ cultivar ‘Yanfu No.10’ lateral buds with 0.2 g/L CYC at three different time points (Figure 1a). With the maturity of axillary buds, the branching promoting effect of CYC was weakened or even disappeared. At A-1, the bud length was significantly different from the control on the 8th day, and the branching phenotype with a shoot length of about 14 mm was easily visible on the 16th day after CYC treatment. At A-2, 16 days after CYC treatment, the shoot length was approximately 12 mm, which was shorter than that at A-1. At A-3, although the bud length was significantly different between CYC treatment and control on the 16th day, no sign of bud outgrowth was observed with a bud length of less than 7 mm (Figure 1b).

### 2.2. Influence of CYC on BRC1 Expression

**Figure 2:**
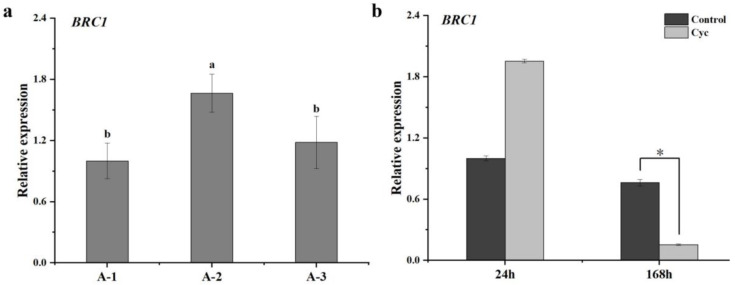
Overview of the *BRC1* expression level using qRT-PCR. (**a**) The *BRC1* expression level among the different maturity state axillary buds at 0 h with A-1, A-2, and A-3 materials (**b**) The *BRC1* expression level after cyclanilide (Cyc) treatment at 24 h and 168 h with A-1 material. Significant differences analysis based on the Tukey test in (**a**) and *t*-test in (**b**) (* *p* < 0.05). Data represent the mean ± SD (*n* = 3).

Considering that *BRC1* plays an important role in bud outgrowth in some plant species, we measured the expression level of *BRC1* (MD06G1211100) among the different maturity state axillary buds at 0 h and after CYC treatment at 24 and 168 h using qRT-PCR. Taking *BRC1* expression level with A-1 material at 0 h as the control, *BRC1* expression increased at A-2, whereas no change was observed at A-3 (Figure 2a). There was an approximately 3-fold decrease in *BRC1* expression compared with the control after CYC treatment at 168 h, whereas no significant change was observed at 24 h (Figure 2b).

### 2.3. Impact of CYC on Phytohormones Concentration

**Figure 3:**
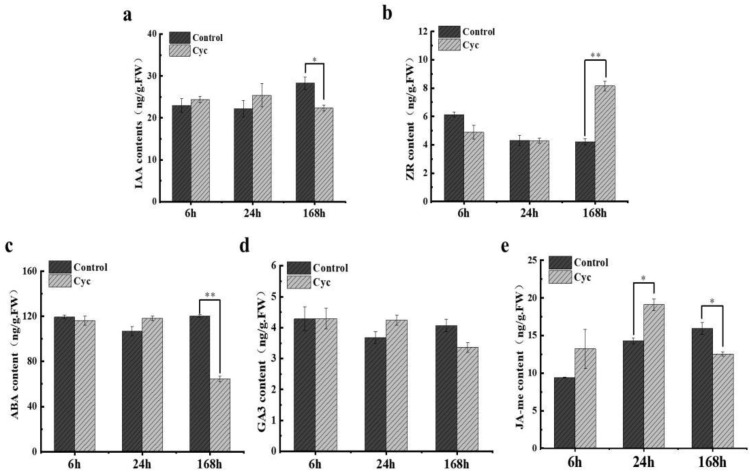
Hormone content in axillary buds at 6–168 h after application of cyclanilide. (**a**) IAA (indole-3-acetic acid) content (**b**) ZR (zeatin riboside) content (**c**) ABA (abscisic acid) content (**d**) GA_3_ (Gibberellin 3) content (**e**) JA-me (methyl jasmonate) content. Cyc: cyclanilide. Data represent the mean ± SD (*n* = 3). Asterisk indicates significant differences based on a *t*-test (*p* < 0.05). Double asterisks indicate extremely significant differences based on the *t*-test (*p* < 0.01).

Since phytohormones play essential roles in bud outgrowth, we analysed the content of phytohormones, including IAA, zeatin riboside (ZR), ABA, GA_3_, and methyl jasmonate (JA-me) after CYC treatment with A-1 material at 6, 24, and 168 h. The results revealed that a marked increase (approximately 2-fold) in the level of ZR and a significant decrease (approximately 2-fold) in the level of ABA were observed at 168 h after CYC treatment compared to the control. A slight decrease in IAA level after CYC treatment, approximately 28 ng/g in the control and 22 ng/g after CYC treatment, was observed at 168 h. No difference in GA_3_ level was observed. The JA-me (methyl jasmonate) level was generally higher after CYC treatment at 24 h, whereas it was higher in the control at 168 h (Figure 3).

### 2.4. Genome-Wide Transcriptional Profiles of Axillary Bud after CYC Treatment

**Figure 4:**
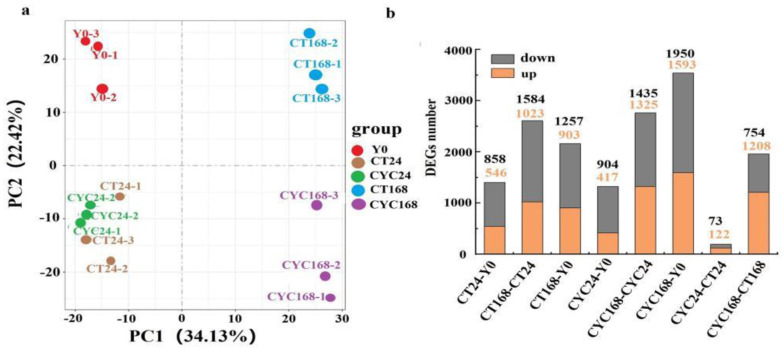
Principle component analysis and differentially expressed genes count. (**a**) Principle component analysis of controls and treatment samples at 0, 24 and 168 h. (**b**) the numbers of differentially expressed genes from all sample comparisons at different time points after treatment.

To investigate the molecular basis and potential pathways related to the promotion of branching by CYC, a transcriptome profiling experiment using RNA-Seq was conducted on axillary buds at 24 and 168 h after CYC treatment with A-1 material. Principle component analysis demonstrated clear differentiation among the control and treatment groups and affirmed clustering of samples from the different time points. Up to 24 h, the samples were not clearly differentiated between the control and treatment groups. However, by 168 h, the two groups were distinct (Figure 4a). The Q30 of the clean reads was between 93% and 93.98% in all the experimental samples. The unique map ratio was between 89.83% and 90.46% in all the collected samples (Supplementary Materials Table S1).

**Figure 5:**
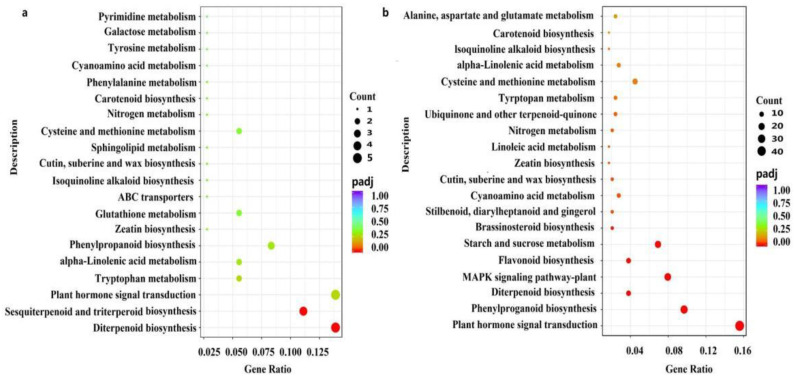
Pathway analysis based on Kyoto encyclopaedia of genes and genomes (KEGG) analysis. (**a**) The KEGG dot map of differentially expressed genes at 24 h after treatment. (**b**) The KEGG dot map of differentially expressed genes at 168 h after treatment.

The total number of lateral bud expressed genes was approximately 30,000 with fragments per kilobase of transcript per million (FPKM) >1.0 in all the experimental samples (Supplementary Materials Excel S1). With the parameters of false discovery rate value <0.01 and the absolute value of log2 ratio >1, there were 1321, 2760, and 3543 differentially expressed genes (DEGs) in the comparisons between CYC24 versus Y0, CYC168 versus CYC24, and CYC168 versus Y0, respectively, where the number indicated the number of hours after treatment. Notably, 195 (73 down and 122 up) and 1962 (754 down and 1208 up) DEGs were significantly enriched in the CYC samples at 24 and 168 h after treatment, respectively (Figure 4b and Supplementary Materials Excel S2). Then, these genes were subjected to the Kyoto encyclopaedia of genes and genomes (KEGG) pathway analysis. At 24 h, the 195 DEGs were significantly enriched in diterpenoid, sesquiterpenoid, and triterpenoid biosynthesis. At 168 h, the 1962 DEGs were significantly clustered into 13 pathways, such as plant hormone signal transduction, starch and sucrose metabolism, and BR and zeatin biosynthesis pathways (Figure 5).

Further, at 168 h, based on the KEGG results, 20 DEGs were clustered in the starch and sucrose metabolism pathways, with trehalose-6-phosphate synthase showing a high expression level after CYC treatment compared with control (Table 1). Overall, six and five DEGs were observed in the BR biosynthesis and zeatin biosynthesis pathways, respectively. BR biosynthesis gene DWF_4_ was up-regulated, whereas BR metabolism gene brassinosteroid-6-oxidase 2 (*BR6OX2*) was down-regulated by CYC. In the zeatin biosynthesis pathway, CTK metabolism genes cytokinin oxidase 1 (*CKX1*) was down-regulated by CYC (Table 2).

To verify the reliability of the RNA-Seq data, five genes related to hormone signalling pathways, cell cycle, and growth were selected randomly in the treatment and control groups for qRT-PCR analysis. The results demonstrated a strong correlation between these two sets of expression data. The correlation indices were both above 90% (Supplementary Materials Table S2).

### 2.5. Weighted Gene Co-Expression Network Analysis of DEGs Produced by CYC Treatment

**Figure 6:**
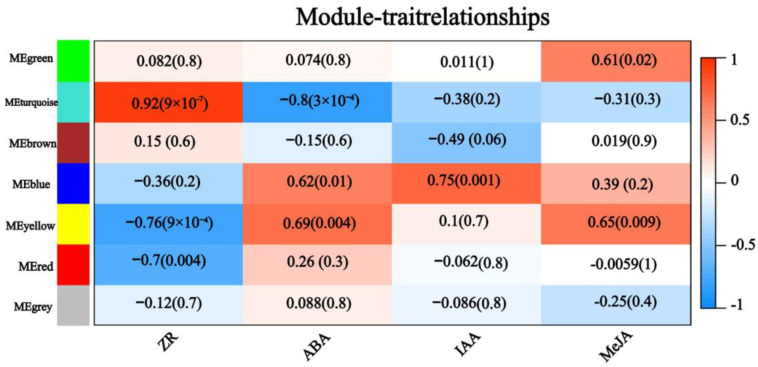
Module-trait associations. Each row corresponds to a module and each column to a trait. Each cell contains the corresponding correlation and *p*-value.

**Figure 7:**
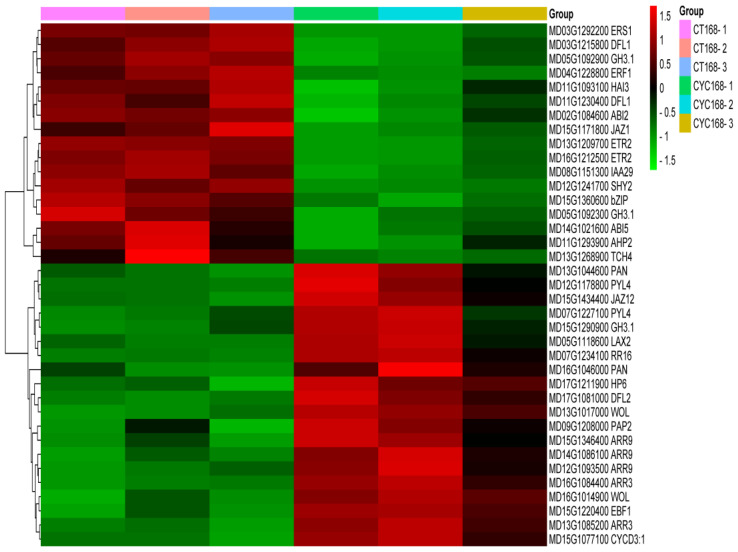
The heatmap of 37 genes involved in the plant hormone signal transduction pathway.

The DEGs produced by CYC treatment were explored using weighted gene co-expression network analysis (WGCNA) as a tool to reveal potentially important pathways. WGCNA produced six modules or clusters containing varying numbers of co-expressed genes. These modules were further carried out in correlation analysis with the four hormones mentioned above, which showed changes after CYC treatment. The turquoise module revealed the highest positive correlation with ZR (r = 0.92) and the highest negative correlation with ABA (r = −0.8) (Figure 6). The turquoise module contained 1259 genes, and 37 genes were significantly enriched in the plant hormone signal transduction pathway by KEGG pathway analysis. Considering the importance of plant hormones in bud outgrowth control, we further analysed these 37 genes. These genes were involved in auxin, CTK, ABA, ethylene, and JA signal transduction pathways. The expression level of these genes changed from log2 ratio = −1 to log2 ratio = 5 between CYC168 vs. CT168 with 20 up-regulated genes and 17 down-regulated genes, such as in the IAA signal transduction pathway. The expression level of a member of the auxin influx carrier genes, such as auxin resistant (*LAX2*), increased with a log2 ratio of 1.3. In the CTK signal transduction pathway, expressions of CTK receptor genes *WOODEN LEG* (*WOL*) and the cytokinin type-A *response regulators* (*RRs*) genes, *ARR3* and *ARR9* were increased. In the ABA signal transduction pathway, ABA signal response genes protein phosphatase 2C genes *ABI2* and *ABI5* were down-regulated in the lateral buds after CYC treatment. (Figure 7).

### 2.6. CTK and ABA Involved in CYC-Regulated Bud Outgrowth

**Figure 8:**
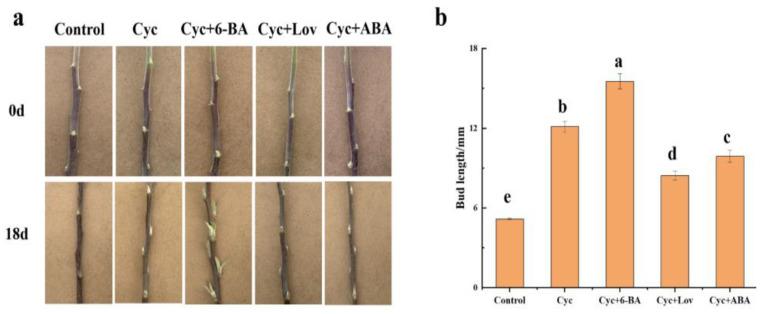
The phenotype and bud length of different pharmacologic treatments. (**a**) The phenotype of different pharmacologic treatments, Scale bar = 5.0 cm. 6-BA (6-benzylaminopurine, a kind of cytokinin). Lov, lovastatin (a synthetic inhibitor of CTK biosynthesis). ABA (abscisic acid). CYC (cyclanilide). (**b**) The bud length of different pharmacologic treatments. Data represent the mean ± SD (*n* = 12). Bars with different letters are significantly different at α = 0.05.

Based on hormonal level and WGCNA analysis, CTK and ABA may play a role in CYC-regulated bud outgrowth. To test this, we supplied 6-benzylaminopurine (6-BA, a synthetic type of CTK), lovastatin (a synthetic inhibitor of CTK biosynthesis) and ABA after CYC treatment to the ‘Yanfu No.10’current-season’s lateral buds. Bud growth was strongly inhibited when lovastatin or ABA was supplied after CYC treatment, whereas 6 BA enhanced the branching effect promoted by CYC. The bud length was approximately 12.1 mm after CYC treatment and 2-fold longer than the negative control (without any plant hormone added), whereas they were approximately 8.4, 9.9, and 15.5 mm by CYC plus lovastatin, CYC plus ABA, and CYC plus 6BA treatment, respectively (Figure 8).

## 3. Discussion

### 3.1. Effect of CYC on Bud Outgrowth in Apple

CYC used alone can decrease apical dominance to induce lateral branching in apple [2,22,23] and in sweet cherry trees [24]. In this study, CYC could promote current-season’s bud branching, with the effect varying with the buds’ mature state (Figure 1), considering that the bud structure, position, and fate generate various branching patterns [25]. Ahmad et al. revealed that temperature caused differential bud outgrowth along with bud positions [26]. Apical dominance involving auxin is the main regulatory factor of lateral bud outgrowth. Burton et al. indicated that CYC could affect polar auxin transport through a mechanism different from other auxin transport inhibitors [1]. At 168 h, the auxin influx carrier gene *LAX2* expression level increased in the lateral buds with CYC treatment (Figure 6), which revealed that CYC might act as an auxin transport inhibitor by first changing the auxin influx level in apple. In a previous study, the auxin efflux carrier genes PIN were proved to be involved in branching control, which acts downstream of CTK, SL, and BRC1 [27,28]. Robert et al. indicated that auxin-dependent cell specification requires balancing both auxin influx and efflux mechanisms [29]. Further studies are warranted to elucidate whether the CYC effect on bud outgrowth has any relation to PIN.

### 3.2. Effect of BRC1 on CYC-Controlled Branching

*BRC1* was considered as an integration of many shoot-branching-related mechanisms, and its expression level was considered to be negatively correlated with branching ability [20]. *BRC1* controls shoot branching by acting downstream of SL, whose expression level is regulated by CTK and SL [30,31,32], and upstream of ABA and PIN [15,27]. Seale et al. showed that *BRC1* expression is unnecessary or sufficient to inhibit bud growth in *Arabidopsis* [32]. Hu et al. revealed that BES1 recruited SMXLs to inhibit *BRC1* expression for branching control [33]. Van Rongen et al. showed that SL-mediated shoot branching control is dependent on connective auxin transport and not on the transcription factor BRC1 [34]. Luo et al. reported that the shoot branching control is dependent or independent on BRC1 [35]. In this study, the *BRC1* expression level was reduced after CYC treatment at 168 h (Figure 2b), which is consistent with the fact that CYC acting as a branching inducer. Among the different maturity state lateral buds, *BRC1* expression tendency (Figure 2a) was not totally consistent with the fact that the branching ability of current-season’s buds weakens with the deepening of maturity state (Figure 1b). These indicated that BRC1 might play an inhibitory role in CYC-induced apple shoot branching, but its role in different maturity state bud branching ability that may be complicated.

### 3.3. Effects of CTK and ABA on CYC-Induced Branching

Prior research demonstrated that CTK stimulates apple branching [36,37]. GA did not affect branching in apple [38]. In this study, CTK level was significantly higher after CYC treatment than control at 168 h. At the same time, no difference in GA_3_ level was observed (Figure 2). CTK was considered as the second positive messenger of branching control [11]. Roma et al. demonstrated that CTKs are initial targets of light in the control of rose bud outgrowth [39]. Xia et al. showed that CTK accumulation was inhibited in response to auxin and SLs, whereas it was increased by sucrose treatment in tomato [17]. Kotov et al. reported that regulation of CTKs in nodes/stems is the centre of external and internal signals [40]. Type-A RRs, which are primary CTK response genes, revealed increased expression level, whereas those of CTK receptor genes *WOODEN LEG* (*WOL*) were both increased after CYC treatment at 168 h (Figure 7). The type-A RRs transcription is rapidly increased by exogenous CTK [41]. UNBRANCHED3 regulated maize and rice branching by modulating the expression of *LONELY GUY1* (*LOG1*) and *type-A RRs* [42]. Further, *CKX 1* was down-regulated by CYC (Table 2). CKX is an enzyme that degrades CTK, reduced expression of *CKX2* caused CTK accumulation and increased the number of reproductive organs in rice [43]. Duan et al. reported that the *OsCKX9 mutant* had significant increases in tiller number [44]. In addition, CYC plus lovastatin could suppress and CYC plus 6 BA enhanced the inducing effort of CYC in shoot branching (Figure 8). It is well confirmed that feedback regulation exists between hormone signalling and hormonal levels for most plant hormones, including CTK [13]. It was observed that CYC treatment indeed increased the content of CTK at 168 h.

ABA is essential for dormancy and plays a vital role in regulating bud outgrowth for the red to far-red light (R: FR) [21,45]. González-Grandío et al. and Zhang et al. revealed that ABA signalling plays a negative role in branching downstream of BRC1 [15,46]. Holalu et al. showed that ABA accumulation in axillary buds works downstream of PIF for shade avoidance responses [47]. In our study, the ABA level was significantly decreased after CYC treatment compared with the control at 168 h (Figure 2). ABA signal response genes *ABI2* and *ABI5* were down-regulated in the lateral bud after CYC treatment (Figure 7). Merlot et al. revealed that ABI1 and ABI2 act in a negative feedback regulatory loop of the ABA signalling pathway. ABA played a negative role in bud outgrowth, and different members of *ABI* had different functions. Further, CYC plus ABA weakened the positive effort of CYC in bud outgrowth (Figure 8) [48]. These results indicate that ABA may work downstream of CYC as a negative agent in bud outgrowth. In summary, the stimulated bud outgrowth by CYC may not be confined to a single hormonal pathway in which CTK and ABA may act as positive and negative agent, respectively, working downstream of CYC.

### 3.4. Other Factors Involved in the Effect of CYC on Branching

Currently, branching outgrowth regulation is a complex network of interacting hormonal, ontogenetic, and trophic signals [7,21,40]. In our study, DEGs after CYC treatment were enriched not only in the hormone pathway but also in other pathways, such as BR biosynthesis and starch and sucrose metabolism pathways (Figure 5). Wang et al. studied the interaction between SL and BR on shoot branching by modulating the MAX2-mediated stability of BZR1 and BES1 [49]. Xia et al. reported that CTK could promote BR synthesis in axillary buds, and BR signalling integrates multiple pathways that control bud outgrowth [17]. In our study, BR biosynthesis gene *DWF4* was up-regulated, whereas BR metabolism gene brassinosteroid-6-oxidase 2 (*BR6OX2*) was down-regulated after CYC treatment (Table 2). CTK may work downstream of CYC. Further studies are warranted to assess whether CTK can influence branching by affecting BR in apple.

Sugars not only provided energy but also acted as a signal for bud outgrowth [50,51]. CTKs up-regulated the genes responsible for the sugar sink strength in previous studies. Glucose activated the synthesis of specific CTK receptors. Sucrose and CTKs could establish directional auxin transport in axillary buds [40]. Patil et al. revealed that sucrose suppressed the inhibitory effect of SL by promoting D53 accumulation [52]. Trehalose-6-phosphate synthase showed a higher expression level after CYC treatment than control (Table 1). CYC is an auxin transport inhibitor [1], and CTK may work downstream of CYC. In addition, there was no sign of bud outgrowth when sugars were exogenously applied to apple as reported in our previous study (data not shown). This suggests that the mode of action of sugars on CYC-stimulated bud outgrowth may be pleiotropic in apple.

## 4. Materials and Methods

### 4.1. Plant Materials

A 1-year-old *Malus* *domestica* Borkh. ‘Yanfu No.10’ (a ‘Fuji’ cultivar)/M26/*Malus robusta* Rehd graft nursery plant was used in this study. The experimental plant was cultivated at a 10 cm × 40 cm spacing in the nursery in Fufeng County, Baoji. The grafting process was as follows: seeds of *M. robusta* Rehd. were sown in 2017. M26 interstocks were grafted onto the *M. robusta* Rehd. in 2018, followed by the grafting of ‘Yanfu No.10’ scions onto the interstocks in autumn of 2019.

Overall, 300 nursery plants with similar current shoot heights (130 cm) were selected for experiment A in June 2020. Further, 60 nursery plants with similar current shoot heights (160 cm) were selected for experiment B. Six nodes of woody stem down the apex of each chosen nursery plants were labelled and treated.

### 4.2. Experimental Arrangements

Experiment A: In this, 300 labelled plants were named A-1, A-2, and A-3 based on different treat times, respectively. A-1 was treated on June 18 (Day 0); A-2 was treated after 5 days (Day 5), and A-3 was treated after 11 days (Day 11) following A-1, respectively (Figure 1). Intact stocks were sprayed once with 0.2 g/L CYC, and controls were sprayed with the exact solutions without CYC. The length of axillary buds was measured every 4 days initiating treatments using a digital calliper. Axillary buds of treated stems were collected for subsequent phytohormones measurement and total RNA extraction with three biological repeats. A single repeat included sampling from 10 plants containing 40–60 axillary buds. The concentration of 0.2 g/L CYC was chosen based on our laboratory research [53]. CYC was dissolved in 0.5% DMSO plus 0.2% TWEEN20.

Experiment B: In this, 5 mM (0.5% DMSO) 6-BA, 5 mM (0.5% DMSO) lovastatin, and 0.02 mM (0.5% DMSO) ABA were supplied 1 day after 0.2 g/L (0.5% DMSO) CYC treatment. The treatments with 0.2 mM (0.5% DMSO) CYC and only 0.5% DMSO represented positive and negative controls, respectively. Each treatment consisted of 12 stocks. The bud length was measured after 18 days after the treatment using a digital calliper.

### 4.3. Hormone Measurements

A total of 0.6 g of fresh axillary buds from A-1 material at 6, 24 and 168 h after CYC treatment were collected for measuring the levels of IAA, ZR, ABA, GA only GA_3_ and MeJA using an ELISA kit (China Agricultural University, Beijing, China) following the manufacturer’s instructions, with three biological replicates. In brief, the samples were ground into powder in liquid nitrogen. The hormones were extracted by overnight incubation in cold 80% (*v*/*v*) methanol with 1 mmol/L butylated hydroxytoluene at 4 °C. After centrifugation at 10,000× *g* for 20 min at 4 °C, two phases were clarified, and the upper phase was collected. Then the extracts were passed through a C18 column and dried under vacuum conditions. The residues were dissolved in PBS (pH7.5) to determine the concentration of IAA, ZR, ABA, GA_3_ and MeJA by the direct ELISA technique. The concentration of the hormones in the samples was obtained by a logit curve, and then the content of the hormones in the samples (ng/g fresh weight) was calculated.

### 4.4. RNA-seq Analysis

Total RNAs were extracted from axillary buds after CYC treatment from A-1 material at 0, 24, and 168 h with the CTAB-based method [54] and used for the RNA-seq analysis with three biological replicates. The libraries were constructed with the Illumina TruSeq kit, and 125 bp single-end reads were obtained from an Illumina Novaseq platform. Reads were mapped to the *Malus domestica* GDDH13 v1.1 genome sequence using Hisat2 v2.0.5. Further, the FPKM fragments mapped reads method was used for calculating expression levels. As previously described, the false discovery rate <0.01 and the absolute value of the log2 ratio >1 were used to identify DEGs. The comparisons of CYC vs. control at the same time point (CYC 24 h vs. CT 24 h, CYC 168 h vs. CT 168) and three different time points in the same treatment (24 h vs. 0 h, 168 h vs. 4 h, and 168 h vs. 0 h) were conducted to identify DEGs. KEGG analyses were performed as previously described [55]. The WGCNA was conducted as described in Langfelder and Horvath (2008) [56].

### 4.5. Quantification of Gene Expression

Total RNAs were extracted from axillary buds after CYC treatment at 0, 24, and 168 h from A-1 material and at 0 h from A-2 and A-3 materials for genes expression analysis, The total RNAs treatment with RNase-free DNase I (Invitrogen, Shanghai, China) removed any residual genomic DNA. The quality of RNAs was determined by running on 2% agarose gel. cDNA was synthesised using a PrimeScript RT Reagent Kit (TaKaRa Bio, Shiga, Japan). Real-time PCR was used for gene expression analysis. The apple *Histone* *H3* gene (MDP0000121016 or MD15G1320600) was used as the internal control base on Guitton et al. (2016) reports [57]. Primers sequences are shown in Supplementary Materials Table S3. The reaction process in the qRT-PCR analysis was the same as previously described [54].

### 4.6. Statistical Analysis

Statistical analysis was performed and graphs were plotted using SPSS version 21.0 (SPSS Inc., Chicago, IL, USA) and Microsoft PowerPoint 2007, respectively. The heatmap was plotted by http://www.bioinformatics.com.cn; accessed on 2 January 2022, an online platform for data analysis and visualisation.

## 5. Conclusions

This study revealed the physiological and molecular mechanisms of CYC inducing bud outgrowth in apple. Hormonal measurement, transcriptome and pharmacologic treatments analysis revealed that the stimulation of bud growth by CYC might involve CTK biosynthesis and signalling, including genes *CKX1* and *ARR3/9*, which provided a direction for further study on the branching promoting mechanism of CYC.

## Figures and Tables

**Table 1:** The differentially expressed genes in the starch and sucrose metabolism pathways at 168 h after cyclanilide treatment.

Gene ID	FPKM		Description
	CYC168	CT168	
MD15G1365900	0.8	6.8	Haloacid dehalogenase-like hydrolase (HAD) superfamily protein
MD15G1326700	1.7	6.8	Haloacid dehalogenase-like hydrolase (HAD) superfamily protein
MD14G1183700	1.7	4.9	Trehalose-phosphatase/synthase 9
MD00G1171800	4.9	14.5	Trehalose-phosphatase
MD13G1089600	3.3	9.7	Trehalose-phosphatase/synthase 7
MD08G1180400	2.8	7.7	Haloacid dehalogenase-like hydrolase (HAD) superfamily protein
MD09G1234800	6.0	14.3	Trehalose-phosphatase/synthase 7
MD15G1223500	40.3	84.2	Sucrose synthase 4
MD06G1237200	0.9	1.8	Sucrose synthase 6
MD09G1192100	4.5	2.2	O-Glycosyl hydrolases family 17 protein
MD14G1004200	4.3	2.0	Glycosyl hydrolase family protein
MD11G1270400	11.5	5.2	Trehalose-6-phosphate synthase
MD13G1186100	5.8	2.5	ADP glucose pyrophosphorylase
MD11G1195800	53.9	23.4	Glycosyl hydrolase family protein
MD15G1113000	6.8	2.9	O-Glycosyl hydrolases family 17 protein
MD11G1178000	20.0	7.8	Glycosyl hydrolase family protein
MD13G1030200	10.8	4.2	O-Glycosyl hydrolases family 17 protein
MD11G1240900	27.9	9.5	Beta glucosidase 46
MD14G1128000	56.4	19.3	Glycosyl hydrolase 9C2
MD10G1316100	64.6	13.2	Glucose-6-phosphate/phosphate translocator 2

FPKM: Fragments Per Kilobase of transcript per Million.

**Table 2:** The differentially expressed genes in the brassinosteroid and zeatin biosynthesis pathways at 168 h after cyclanilide treatment.

Gene ID	FPKM		Description	Pathway
	CYC168	CT168		
MD13G1058000	0.8	2.7	Cytochrome P450 superfamily protein	BR biosynthesis
MD17G1064800	1.9	3.9	Brassinosteroid-6-oxidase 2	BR biosynthesis
MD06G1146700	8.2	21.0	Cytochrome P450 superfamily protein	BR biosynthesis
MD06G1146000	23.0	46.9	Cytochrome P450 superfamily protein	BR biosynthesis
MD17G1167200	6.7	3.2	Cytochrome P450 superfamily protein	BR biosynthesis
MD17G1120200	1.6	0.0	Cytochrome P450 superfamily protein DWF4	BR biosynthesis
MD14G1078600	0.5	6.1	Cytokinin oxidase/dehydrogenase 1	Zeatin biosynthesis
MD16G1041700	0.9	2.8	Isopentenyl transferase 1	Zeatin biosynthesis
MD03G1267900	2.6	6.3	Isopentenyl transferase 3	Zeatin biosynthesis
MD17G1076700	1.9	0.8	Cytochrome P450, family 735, subfamily A, polypeptide 1	Zeatin biosynthesis
MD15G1208300	4.0	0.5	Cytokinin oxidase/dehydrogenase 3	Zeatin biosynthesis

FFPKM: Fragments Per Kilobase of transcript per Million.

## Data Availability

All data supporting the reported results are included within the article or its Supplementary Materials Files.

## Supplementary Materials

The following are available online at https://www.mdpi.com/article/10.3390/ijms23020581/s1.
