# Supplementary material for: Cyclanilide Induces Lateral Bud Outgrowth by Modulating Cytokinin Biosynthesis and Signalling Pathways in Apple Identified via Transcriptome Analysis
Source: Int J Mol Sci. 2022 Jan 6;23(2):581. doi: 10.3390/ijms23020581 (PMC8776233; doi:10.3390/ijms23020581)
Supplement: Supplementary file 1 [file ijms-23-00581-s001.zip › Table S1.pdf]

sample	clean_reads	Q30	unique_map
Y0_1	43138052	93.47	38751407(89.83%)
Y0_2	44261848	93.92	40001261(90.37%)
Y0_3	43101352	93.76	38798032(90.02%)
CT24_1	44169246	93.79	39877337(90.28%)
CT24_2	45813952	93.31	41419384(90.41%)
CT24_3	41927590	93.98	37929298(90.46%)
CYC24_1	43654872	93.66	39421676(90.3%)
CYC24_2	42451212	93	38345321(90.33%)
CYC24_3	42495302	93.71	38414928(90.4%)
CT168_1	44955050	93.65	40580851(90.27%)
CT168_2	43025940	93.58	38878452(90.36%)
CT168_3	44053172	93.85	39578634(89.84%)
CYC168_1	40611590	93.52	36634822(90.21%)
CYC168_2	40757694	93.67	36666236(89.96%)
CYC168_3	43876572	93.76	39535547(90.11%)
