# Supplementary material for: Cyclanilide Induces Lateral Bud Outgrowth by Modulating Cytokinin Biosynthesis and Signalling Pathways in Apple Identified via Transcriptome Analysis
Source: Int J Mol Sci. 2022 Jan 6;23(2):581. doi: 10.3390/ijms23020581 (PMC8776233; doi:10.3390/ijms23020581)
Supplement: Supplementary file 1 [file ijms-23-00581-s001.zip › Table S2.pdf]

Gene name	Gene locus	CT24		Cyc24		CT168		Cyc168	
		RNA-seq	qPCR	RNA-seq	qPCR	RNA-seq	qPCR	RNA-seq	qPCR
MdCYCD3	MD15G1077100	4.83	1	2.96	5.57	2.22	17.92	5.07	7.32
MdABI2	MD02G1084600	7.41	5.79	12.92	9.59	14.54	30.99	4.99	7.62
MdTCH4	MD13G1268900	3.72	12.88	3.20	11.03	8.63	26.63	3.08	7.90
MdPP2C	MD07G1291000	5.96	0.17	8.53	1.06	9.06	1.46	3.91	0.99
MdAHP1	MD12G1226800	185.34	650.97	286.59	2929.61	107.59	2929.61	86.75	465.43
