# Supplementary material for: Cyclanilide Induces Lateral Bud Outgrowth by Modulating Cytokinin Biosynthesis and Signalling Pathways in Apple Identified via Transcriptome Analysis
Source: Int J Mol Sci. 2022 Jan 6;23(2):581. doi: 10.3390/ijms23020581 (PMC8776233; doi:10.3390/ijms23020581)
Supplement: Supplementary file 1 [file ijms-23-00581-s001.zip › Table S3.pdf]

Gene name	GeneID	Primer sequence（5'-3'，F，R）
BRC1	MD06G1211100	AGCCCAGCAAAACCGTGGAGT<br>TGGAGCCTCATCGACGCCTG
MdCYCD3	MD15G1077100	TGCCAAAGTTGAGGAGACCC<br>TAGGGTTCTGGTTGAGCTTCC
MdABI2	MD02G1084600	GGATTTAGCTCAGGCAGTTGCT<br>TTTGAACCGGAACCTGCGAG
MdTCH4	MD13G1268900	TACTTGCGCTCAGAAGGGTC<br>GGTCGCCACTAAGATTCCCC
MdPP2C	MD07G1291000	GGGTACGGCGTTGTATCATT<br>ACACGGTTTCCGTTCCAAGT
MdAHP1	MD12G1226800	AACGTCGAAGGGTGCGTAAAA<br>CCTTGGAAGCGAACTGCCG
HistoneH3	MD15G1320600	ATGGCCCGTACCAAGCAA<br>CGGATTTCACGAAGAGCAAC
